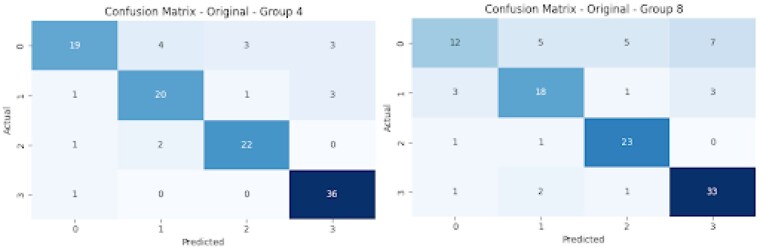# 692 Enhancing Burn Patient Discharge Predictions: Random Forest and Logistic Regression with Six-Clicks Oversampling

**DOI:** 10.1093/jbcr/iraf019.321

**Published:** 2025-04-01

**Authors:** Abhinav Karnati, Douglas Bettarelli, Vishal Bandaru, Kurt Grabow, Khaja Siddiqui, Mark Gao, Rafael Cacao, Senja Collins, Chip Shaw, Alan Pang, John Griswold

**Affiliations:** Texas Tech University Health Sciences Center; Texas Tech University Health Sciences Center; Texas Tech University Health Sciences Center; Texas Tech University Health Sciences Center School of Medicine; Texas Tech University Health Sciences Center; Texas Tech University Health Sciences Center; Texas Tech University; Trustpoint Rehabilitation Hospital of Lubbock; Texas Tech University Health Sciences Center; Texas Tech University Health Sciences Center School of Medicine; Texas Tech University Health Sciences Center School of Medicine

## Abstract

**Introduction:**

Predicting discharge disposition for burn patients with large total body surface area (TBSA) is essential to optimizing care and improving long-term outcomes. Patient recovery, expectations, and goal setting require early physician planning, but recovery is rarely based solely on TBSA. A multidisciplinary approach, paired with simple yet comprehensive assessment tools, can enhance patient care discussions and family preparedness. The Activity Measure for Post-Acute Care Six-Clicks, a functional screening tool used by physical therapists in hospital settings, has shown promise in predicting discharge disposition and can significantly improve treatment planning, resource allocation, and the overall patient experience in burn care.

**Methods:**

Patient data collected to predict discharge disposition included demographics, TBSA, and Six-Clicks scores. The original dataset had 238 patients, which was expanded to 580 total samples using SMOTE to address class imbalance. Oversampling ensured uniform distribution across the four discharge disposition categories: home discharge, skilled nursing facilities/long-term care, transfers to another institution, and expired/hospice. Logistic regression and Random Forest models were trained using feature sets. Group 4 and Group 8 included predictors such as age, TBSA, intubation status, and inpatient surgeries, with Group 4 additionally incorporating the Six-Clicks scores to assess its predictive value. Models were evaluated using accuracy, ROC AUC, and confusion matrices.

**Results:**

Six-Clicks scores as a feature in Group 4 significantly improved the prediction of discharge disposition shown in the confusion matrix. It exhibited better classification across the four discharge categories, with more correct predictions than Group 8, particularly in categories representing higher mobility impairments.

**Conclusions:**

Group 4 significantly improved model predictions, emphasizing that adding the Six-Clicks score to existing assessment criteria such as TBSA can better evaluate discharge disposition and improve treatment plan effectiveness.

**Applicability of Research to Practice:**

Enhancing prediction accuracy has far-reaching impacts beyond the hospital. It improves the coordination and effectiveness of multidisciplinary teams by ensuring the appropriate level of care is provided. Including a tool such as AM-PAC Six-Clicks improves discharge planning accuracy, leading to more efficient management of healthcare resources and better patient outcomes.

**Funding for the Study:**

No external funding was provided for this study.